# A cross-category puffing topography, mouth level exposure and consumption study among Italian users of tobacco and nicotine products

**DOI:** 10.1038/s41598-019-55410-5

**Published:** 2020-01-08

**Authors:** Joshua Jones, Sandra Slayford, Adam Gray, Kathryn Brick, Krishna Prasad, Christopher Proctor

**Affiliations:** British American Tobacco, Group Research and Development, Regents Park Road, Southampton, SO15 8TL UK

**Keywords:** Human behaviour, Human behaviour, Analytical chemistry, Analytical chemistry, Optical sensors

## Abstract

Actual use studies play a key part in evaluating the reduced risk potential of tobacco and nicotine products. This study was undertaken to determine the puffing topography, mouth level exposure (MLE) and average daily consumption (ADC) relating to two commercially available tobacco heating products (THPs) and a prototype electronic cigarette (or e-cigarette) among Italian non-mentholated 7 mg ISO tar cigarette smokers. The study was conducted in Milan, Italy, with three groups of approximately 50 participants. Groups 1 and 3 included adult smokers of 7 mg ISO tar tobacco cigarettes, and Group 2 consisted of both solus vapers and dual users of vapour and tobacco products. Amongst smokers, e-cigarette mean puff volumes (41.6 mL vs 41.3 mL) and mean puff durations (1.4 s vs 1.5 s) were similar to that of the cigarette, although the average usage session was significantly longer (1064.8 s vs 289.5 s) with a higher total number of puffs (58.6 vs 17.3), however this may be attributable to differences in product operation. There were no significant differences across puffing topography measurements observed between smokers (Group 1) and regular vapers/dual users (Group 2) when using the e-cigarette. As consistent with previous research, users took, on average, larger mean puff volumes when using a THP compared to the reference cigarette (C651), although puff numbers and puff durations remained similar. The average interval between puffs was considerably shorter for THP1.0(T) compared to THS2.4(T) (11.0 s vs 17.1 s). MLE to nicotine-free dry particulate matter and nicotine was significantly reduced for THP1.0(T) and THS2.4(T) compared to the tobacco cigarette (C651). MLE to nicotine was also significantly reduced for the e-cigarette (IS1.0(T)) compared to C651. The average daily consumption (ADC) of cigarettes by groups 1 and 3 were higher than the respective ADCs of both THP consumables. There were no significant differences in ADC when comparing the same product between different groups. Differences seen between sensory scores for each of the product categories may be attributed to fundamental differences in design and mode of operation resulting in very different characteristics of the aerosol generated.

## Introduction

With an estimated 1.1 billion smokers worldwide as of 2016^1^, the use of combustible tobacco products has been identified as one of the leading preventable causes of human disease^2^. Most of these smoking-related diseases – such as cardiovascular disease, respiratory disorders and lung cancer – are attributed to the inhalation of toxicants within tobacco smoke^3^, released when the tobacco is combusted^4,5^. As these toxicants are often the product of combustion and pyrolysis, recent advances have focused on heating (at temperatures below 350 °C^6^) rather than burning tobacco, or removing tobacco exposure altogether in the form of electronic cigarettes (also known as e-cigarettes, or Electronic Nicotine Delivery Systems (ENDS)). Based on current available evidence, Public Health England^7^ have supported the statement that using e-cigarettes is likely to be around 95% less harmful than smoking cigarettes and concluded that “switching completely from smoking to vaping conveys substantial health benefits”.

With regards to tobacco heating products (THPs), the commercially available glo™ (THP1.0), has been shown to produce an aerosol containing at least a 90% lower concentration of TobReg 9 toxicants (priority cigarette smoke toxicants for reduction, highlighted by WHO Tobacco Product Regulation Study Group) than conventional cigarette smoke under laboratory conditions^8^. Furthermore, a study looking at multiple THPs confirmed that levels of key carcinogens were significantly reduced in THP emissions compared to conventional tobacco cigarettes^9^, and an independent review by the Committees on Toxicology, Carcinogenicity and Mutagenicity of Chemicals in Food, Consumer Products and the Environment^10^ concluded that there is likely to be a reduction in overall risk for smokers switching to THPs.

In addition to assessing the emissions of these products, it is important to understand if consumers are using these products in a manner that supports their potential reduction in overall toxicant exposure compared with smoking cigarettes. ‘Actual use’ studies therefore play a key part in the proposed scientific framework^11,12^ for evaluating the reduced risk potential of tobacco and nicotine products. As stated within the Modified Risk Tobacco Product Application (MRTPA) guidelines, the US Food and Drug Administration (FDA) have recommended conducting ‘actual use’ studies, both pre- and post-product launch, that allow consumers to interact freely with the products in “real-world conditions”^13^.

This paper describes an ‘actual use’ study undertaken with the aim of understanding consumer behaviour relating to THPs (THP1.0(T) and THS2.4(T)) and an e-cigarette, compared with a conventional tobacco cigarette (C651). A central location test was conducted to measure puffing topography and mouth level exposure (MLE) among Italian consumers of e-cigarettes, THPs and conventional tobacco cigarettes.

In the past, research into the MLE of conventional tobacco cigarettes has been widely based around the relationship between the materials trapped within the filter and user’s mouth level exposure to smoke^14–17^. Using this relationship, researchers are able to collect used cigarette filters from participants and analyse them to estimate mouth level exposure for a particular product. There are fundamental differences in the function of the mouth-end sections between THPs and cigarettes, meaning additional research is necessary before MLE from THPs can be estimated using this approach. Furthermore, as e-cigarettes do not contain filters like cigarettes, the part-filter method for estimating MLE is inappropriate.

This study utilised a real-time optical obscuration technique, previously described by Gee *et al*.^18^ and Slayford & Frost^19^, to estimate MLE to nicotine free dry particulate matter (NFDPM) and nicotine from THPs (THP1.0(T) and THS2.4(T)) and a cigarette (C651). For the e-cigarette (IS1.0(T)), MLE to ‘aerosol collected mass’ (ACM) was estimated using the relationship between aerosol exposure and the ‘device mass loss’ (DML) after each use session. ACM is the total mass of compounds collected on the Cambridge filter pad after each machine usage cycle, including both water and nicotine.

Measuring MLE and puffing topography across a range of product categories will enable the placement of tobacco and nicotine products on a risk continuum and aid the scientific assessment of these products relative to individual and population risk^11^.

## Methods

### Study products

**Figure 1:**
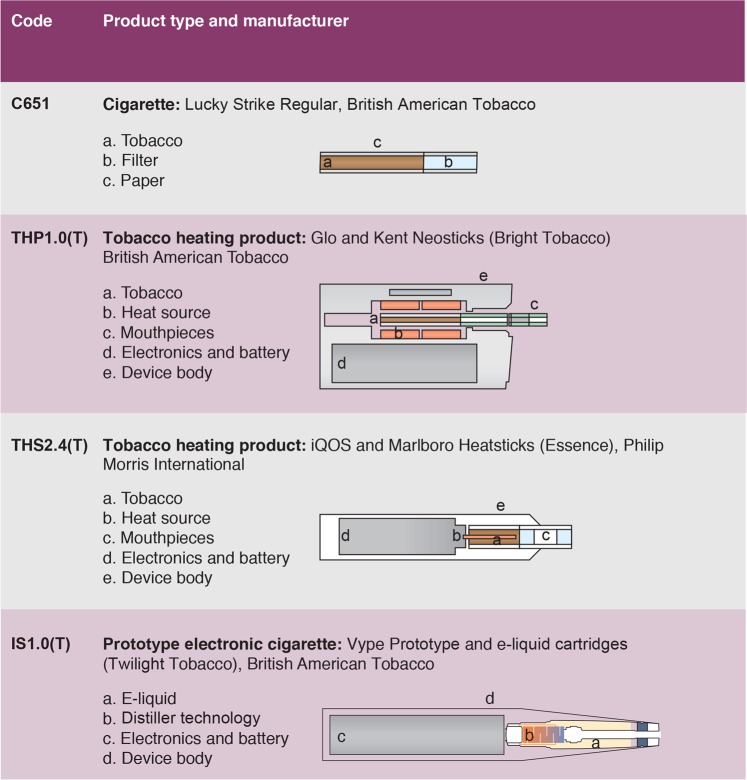
Study product descriptions.

A total of four products, ranging across three product categories, were evaluated in this study (Fig. 1).

#### Tobacco heating devices

THP1.0(T) consists of the commercial glo™ (British American Tobacco (BAT)) heating device with Bright Tobacco Kent Neostiks^™^. It is, in brief, a handheld electronic device that produces an inhalable aerosol by heating a specific tobacco consumable to a maximum temperature of 240 °C ± 5 °C^6^. Similarly, THS2.4(T) is the commercially available iQOS™ (Philip Morris SA Neuchatel, Switzerland) tobacco heating system, used with an Essence tobacco HeatStick^™^ (Ex. Italy) consumable. Both devices rely on different mechanisms for heating the tobacco with THS2.4(T) having a higher operating temperature (up to approx. 350 °C), however the basic principle of operation is similar^6,20^.

#### Electronic cigarette

Participants were also provided with the most recent version of a Vype**™** prototype 10-watt rechargeable e-cigarette (IS1.0(T)) device developed by BAT. This device utilises distiller technology for both the heating and wicking of the e-liquid, rather than a traditional coil and wick system. This technology relies on a heated stainless-steel mesh for both capillary liquid transfer and vaporisation. Upon button activation, the device provides an electrical current to the heating element contained within the disposable cartridge containing e-liquid, producing an inhalable aerosol.

A 2 mL ‘Twilight Tobacco’ flavoured e-liquid cartridge was used for this study, containing 5 mg/mL nicotine and a vegetable glycerol (VG): propylene glycol (PG) ratio of approximately 60:40.

#### Conventional tobacco cigarette

A non-mentholated commercially available tobacco cigarette Lucky Strike (C651) yielding 7 mg ISO^21^ NFDPM was used in the study.

### Study participants & ethics statement

The study was conducted in Milan, Italy, with three groups of approximately 50 participants. They were recruited by an independent market research agency following the International Code on Market Opinion and Social Research and Data Analytics^22^.

Groups 1 and 3 comprised adult Italian smokers of 7 mg ISO NFDPM non-mentholated cigarettes between the ages of 25 years and 7 months and 64 years. Participants were eligible for inclusion within this study group if they smoked eight or more cigarettes per day and had been smoking for more than seven years.

Group 2 consisted of adult Italian vapers and dual users of vapour and tobacco products between the ages of 25 years and 7 months and 64 years. Participants were eligible for inclusion if they were vaping at least once per day and had been vaping for more than 6 months.

All participants were screened using a questionnaire and provided informed consent prior to participation. Females were excluded if they reported that there was a possibility they could have been pregnant during recruitment. Participants were informed that they were free to withdraw from the study at any time and received pro-rata remuneration for their involvement in the study.

The study protocol and Informed Consent Form were approved by the Human Research Committee (HRC), British American Tobacco’s internal committee dedicated to ensuring all studies involving human subjects are carried out in accordance with the ethical principles outlined in the Declaration of Helsinki and other relevant guidelines (HRC_SPS_16_099).

### Study design

#### Initial visit to central study location

Before initiation of the study, volunteers were requested an initial visit to the central study location. Here, volunteers received detailed information about the study and were then invited to sign an informed consent form relevant to their recruited user group. Once participation in the study had been confirmed, volunteers were provided with a randomly allocated study product (alongside any associated consumables) and a consumption diary. The supply of product for each 5-day home placement was equivalent to five times the reported daily consumption of cigarettes, but no more than 100 cigarettes/THP consumables per day or two e-cigarette consumables per day.

#### Home use

Volunteers were asked to take home and use the allocated study product in place of their normal product for five days. They were asked to record numbers of study and non-study cigarettes or consumables used each day in the consumption diary provided.

#### Further central location tests

On day five, volunteers were asked to revisit the central study location to return their consumption diaries and undertake further assessments. They completed a sensory questionnaire from memory (see Section 3.6), under the supervision of agency staff, relating to the product for which they had completed the 5-day home placement. Participants were asked to score various product-related sensory attributes on both magnitude and ‘just-right’ scales.

Volunteers were then asked to use one sample of the allocated study product *ad libitum* through a modified Smoking Analyser 7 (SA7) unit^23^, as described in Section 2.4, to gather puffing topography and optical obscuration data for each product-use session. This process was repeated on future visits, ensuring measurements were taken across all study products placed within the groups (see section 2.3.4).

#### Distribution of study products

Group 1 volunteers used IS1.0(T), THS2.4(T) and C651 for 5 days each, across a total of four visits to the central location.

Group 2 used the IS1.0(T) only, and therefore visited the central location twice.

Group 3 volunteers used THP1.0(T) and C651 for 5 days each, across a total of three visits.

### Analytical methods

#### Puffing topography

For each product use session, puffing topography measurements were taken to record puff volumes, puff durations, puff intervals, the number of puffs and the total session length. Participants were asked to use each product twice, through a puffing topography device, with an interval of 20 minutes between each replicate. The maximum session length for product use was set at 20 minutes, however users of C651, THP1.0(T) and THS2.4(T) were limited to one stick/cigarette per session.

**Figure 2:**
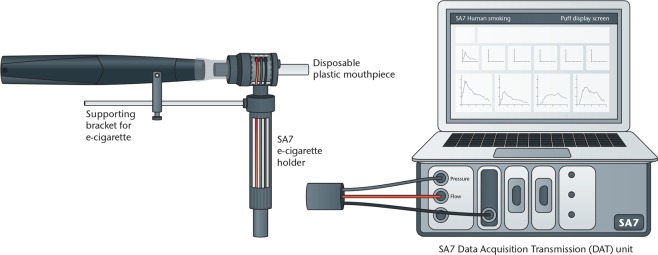
Illustration of SA7 puffing topography device with modified product holder.

Data were recorded using a bespoke SA7 portable topography analyser (Fig. 2), consisting of a product holder connected to a data acquisition and transmission unit (DAT) with two unidirectional pressure transducers placed on either side of an orifice. The pressure transducers work by detecting a pressure change across the orifice (2 mm in diameter), which is proportional to the flow rate squared^9^. The product holder was modified for use with e-cigarettes as outlined by Cunningham *et al*.^23^ (Fig. 2).

#### Mouth level exposure

For the THPs and cigarette, MLE to NFDPM and nicotine was estimated using a real-time measurement of optical obscuration for each puff of aerosol or smoke. In brief, the optical obscuration methodology^19^ works on the principle that light from an LED within the device head is obscured by aerosol generated when puffing on a product. The extent of this optical obscuration is correlated against mainstream NFDPM exposure for a specific product using machine smoking across a spectrum of pre-set puffing regimes (Supplementary Tables S1–S3). The amounts of water and nicotine in the mainstream aerosol were determined by capturing the total particulate matter (TPM) on a Cambridge filter pad using a PM1 smoking machine (Borgwaldt KC, Hamburg, Germany) and subsequently analysing the extracted TPM by gas chromatography. The range of calculated NFDPM (NFDPM = TPM − nicotine − water) values were then used to determine the most appropriate factors for calculation of ‘optical NFDPM’^19^. Estimates of MLE to nicotine were generated using the relationship between NFDPM and nicotine, established using calibration graphs. These relationships allowed MLE to nicotine to be estimated using ‘optical NFDPM’ in place of NFDPM. The water analysis for all products was based on those outlined in the International Standard ISO 4387, however it should be noted that this method may not account for all water released in high water-content aerosols, leading to an overestimation of NFDPM^24^.

As it was discovered that puff interval has a significant effect on the relationship between NFDPM and nicotine for C651, and likely other tobacco cigarettes, a number of regimes with shorter 10-second puff intervals were included across the calibration smoking **(**Supplementary Table S3**)**. This effect is likely due to lower puff intervals leading to increased combustion of the cigarette – increasing/decreasing the ratio of NFDPM to nicotine in the captured aerosol. No such effect was observed with the other study products and so a constant 30-second puff interval was used for these calibrations.

For the e-cigarette (IS1.0(T)), the relationships between device mass loss (DML) and ACM and nicotine were used for MLE estimation. DML was measured by weighing the device pre- and post-use (maximum of 5 minutes between end of usage session and weighing of device) and then calculating the difference in mass. As with the optical obscuration approach, a number of smoking regimes were used to produce calibration graphs and the amounts of ACM and nicotine were measured using a gas chromatography methodology.

*Mean MLE* and *average daily consumption (ADC)* were used to calculate *MLE per day*.

#### Verification of optical estimates

The use of optical obscuration as an appropriate measure of MLE to NFDPM and nicotine was verified by establishing correlations between the NFDPM collected from mainstream aerosol and the associated optical reading (OR) taken as the aerosol passes through the head of the puffing topography device. Using a purpose-built program within the SA7 software suite, a selection of participants’ topography records from the field study were duplicated using a 4-port linear smoking machine (LM4X, Borgwaldt KC) in the laboratory connected to an SA7.

A total of 30 topography records were selected for each product used. For THP1.0(T), THS2.4(T) and C651, five records were selected at low, medium and high total ‘optical NFDPM’, with a further five records being selected at low, medium and high mean puff volumes. The same principle was applied for IS1.0(T), however due to operational differences with this device, 15 records were selected over a range of puff durations rather than volumes, and an additional 15 selected over a range of DML values rather than ‘optical NFDPM’.

The mainstream aerosol generated by each product was collected on a Cambridge filter pad and analysed using gas chromatography for water and nicotine. The mainstream NFDPM was then calculated using the TPM, water and nicotine values, and correlated against the ‘optical NFDPM’ or DML for each product.

### Statistical analysis

Statistical analysis was carried out using SAS version 9.4 statistical software (SAS Institute Inc., Cary, NC, USA). Puffing topography, MLE, ADC and sensory data are presented in the summary tables as mean values ± standard deviation (SD). Where data was not normally distributed, log transformations were applied before analysis. A linear mixed model (Proc Mixed) was used to analyse for variance within the user groups. If a significant difference was found between the means, Tukey’s post-hoc test was applied to examine the source of the difference. Inter-group differences were also analysed using the same approach where the same product was used by multiple groups. For comparison of sensory ‘just right’ scores, Student’s t test was used to analyse for differences between mean scores and the ‘just right’ score of 3.

## Results

### Study participants

Three groups of participants (Table 1) were recruited for the study, with between 50 and 52 participants in each group. Groups 1 and 3 were composed of tobacco cigarette smokers, whereas Group 2 consisted of e-cigarette users.

**Table 1:** Demographic characteristics of study participants.

*Number of participants*	Group 1	Group 2	Group 3
	50	50	52
***Gender***			
Male	**24** (48%)	**26** (52%)	**26** (50%)
Female	**26** (52%)	**24** (48%)	**26** (50%)
***Number per age-group***			
25–29 years	**6** (12%)	**9** (18%)	**6** (12%)
30–44 years	**19** (38%)	**28** (56%)	**20** (38%)
45–65 years	**25** (50%)	**13** (26%)	**26** (50%)

The demographics of participants within Groups 1 and 3 were very similar, however Group 2 contained a significantly higher proportion of participants in the 25–29 (18% vs 12%) and 30–44 (56% vs 38%) year age ranges.

Gender distributions within all groups were approximately equal (48–52%).

### Puffing topography

Mean values and standard deviations for puff number, total puff volume, mean puff volume, puff duration, puff intervals and overall time taken to complete the session are shown in Table 2.

**Table 2:** Comparison of puffing topography data between products within a user group.

User group	Product	Puff number (n)^a^		Total puff volume (mL)^a^		Mean puff volume (mL)		Puff duration (s)		Puff interval (s)		Session Length (s)*	
		Mean (±SD)	Tukey’s ranking^b^	Mean (±SD)	Tukey’s ranking^b^	Mean (±SD)	Tukey’s ranking^b^	Mean (±SD)	Tukey’s ranking^b^	Mean (±SD)	Tukey’s ranking^b^	Mean (±SD)	Tukey’s ranking^b^
**1** n = 50	**C651**	**17.3** (**±**5.9)	**b**	**682.8** (**±**224.7)	**b**	**41.3** (**±**12.7)	**b**	**1.5** (**±**0.5)	**a**	**18.8** (**±**10.6)	**b**	**289.5** (**±**85.7)	**b**
	**THS2.4(T)**	**15.0** (**±**5.5)	**b**	**730.9** (**±**350.4)	**b**	**48.8** (**±**17.9)	**a**	**1.4** (**±**0.5)	**a**	**17.1** (**±**20.7)	**b**	**195.6** (**±**87.2)	**c**
	**IS1.0(T)**	**58.6** (**±**37.6)	**a**	**2347.2** (**±**1480.7)	**a**	**41.6** (**±**16.0)	**b**	**1.4** (**±**0.6)	**a**	**27.6** (**±**22.5)	**a**	**1064.8** (**±**104.8)	**a**
**2** n = 50	**IS1.0(T)**	**63.6** (**±**36.2)	—	**2503.1** (**±**1629.4)	—	**41.2** (**±**17.0)	—	**1.5** (**±**0.6)	—	**23.3** (**±**17.3)	—	**1030.7** (**±**88.8)	—
**3** n = 52	**C651**	**16.0** (**±**5.6)	**a**	**596.8** (**±**197.1)	**a**	**39.3** (**±**12.4)	**b**	**1.6** (**±**0.5)	**a**	**18.8** (**±**10.6)	**a**	**269.3** (**±**88.0)	**a**
	**THP1.0(T)**	**15.4** (**±**7.4)	**a**	**731.3** (**±**437.6)	**a**	**46.6** (**±**16.8)	**a**	**1.6** (**±**0.5)	**a**	**11.1** (±5.8)	**b**	**150.4** (±40.5)	**b**

^a^C651, THS2.4(T) and THP1.0(T) are stick-based products and therefore had a limited usage period compared to IS1.0(T).

^b^Different letters within a group indicate statistically significant differences (p < 0.05) between mean values.

Group 1 participants took significantly more puffs on the IS1.0(T) compared to C651 and THS2.4(T), resulting in much higher total puff volumes. The mean puff volumes were similar for the IS1.0(T) and C651, but users took larger mean puff volumes when using the THS2.4(T). The average puff durations for the three products were very similar, however the interval between puffs was significantly longer for the IS1.0(T).

On average, Group 3 participants took similar numbers of puffs when using the C651 and THP1.0(T), however the total and mean puff volumes were significantly higher for the THP1.0(T). The interval between puffs for the THP1.0(T) was also much lower than that of the C651, with overall shorter usage sessions.

No significant differences were observed when comparing the puffing topography across user groups (Table 3)

**Table 3:** Comparison of puffing topography data between groups.

Product	User group	Puff number (n)		Total puff volume (mL)		Mean puff volume (mL)		Puff duration (s)		Puff interval (s)		Session Length (s)	
		Mean (±SD)	Pr > (t)	Mean (±SD)	Pr > (t)	Mean (±SD)	Pr > (t)	Mean (±SD)	Pr > (t)	Mean (±SD)	Pr > (t)	Mean (±SD)	Pr > (t)
C651	**1**	**17.3** (**±**5.9)	**0.2438**	**682.8** (**±**224.7)	**0.0541**	**41.3** (**±**12.7)	**0.4294**	**1.5** (**±**0.5)	**0.2308**	**18.8** (**±**10.6)	**0.9891**	**289.5** (**±**85.7)	**0.2084**
	**3**	**16.0** (**±**5.6)		**596.8** (**±**197.1)		**39.3** (**±**12.4)		**1.6** (**±**0.5)		**18.8** (**±**10.6)		**269.3** (**±**88.0)	
IS1.0(T)	**1**	**58.6** (**±**37.6)	**0.4258**	**2347.2** (**±**1480.7)	**0.6135**	**41.6** (**±**16.0)	**0.8594**	**1.4** (**±**0.6)	**0.3305**	**27.6** (**±**22.5)	**0.3051**	**1064.8** (**±**104.8)	**0.0996**
	**2**	**63.6** (**±**36.2)		**2503.1** (**±**1629.4)		**41.2** (**±**17.0)		**1.5** (**±**0.6)		**23.3** (**±**17.3)		**1030.7** (**±**88.8)	

Upon comparison of THP1.0(T) use in Group 3 and THS2.4(T) in Group 1, the only clear difference between usage patterns related to the relatively short puff interval observed with the THP1.0(T).

### Mouth level exposure (MLE)

MLE data is summarised in Table 4 by study product and user group. The MLEs to NFDPM and nicotine *per session* and *per day* were significantly lower for both THPs compared to the reference cigarette. Average percentage reductions in MLE per usage session compared to C651 were approximately −49% NFDPM and −38% nicotine for THS2.4(T), and −72% NFDPM and −79% nicotine for THP1.0(T).

**Table 4:** Comparison of MLE to NFDPM/ACM and nicotine between products within a user group.

User group	Product	MLE to NFDPM (mg/session)		MLE to ACM (mg/session)		MLE to nicotine (mg/session)		MLE to NFDPM (mg/day)		MLE to ACM^a^ (mg/day)		MLE to nicotine^b^ (mg/day)	
		Mean (±SD)	Tukey’s ranking^c^	Mean (±SD)	Tukey’s ranking^c^	Mean (±SD)	Tukey’s ranking^c^	Mean (±SD)	Tukey’s ranking^c^	Mean (±SD)	Tukey’s ranking^c^	Mean (±SD)	Tukey’s ranking^c^
**1**	**C651**	**19.0** (**±**7.7)	**a**	—	—	**1.55** (**±**0.63)	**a**	**256.4** (**±**150.4)	**a**	—	—	**20.9** (**±**12.3)	**a**
	**THS2.4(T)**	**9.6** (**±**5.0)	**b**	—	—	**0.98** (**±**0.51)	**b**	**89.9** (**±**84.6)	**b**	—	—	**9.1** (**±**8.6)	**b**
	**IS1.0(T)**	—	—	**180.1** (**±**137.8)	—	**0.69** (**±**0.53)	**c**	—	—	**1883.1** (**±**897.6)	—	**7.3** (**±**3.5)	**b**
**2**	**IS1.0(T)**	—	—	**209.2** (**±**157.4)	—	**0.80** (**±**0.60)	—	—	—	**1914.2** (**±**679.4)	—	**7.4** (**±**2.6)	—
**3**	**C651**	**16.7** (**±**7.6)	**a**	—	—	**1.36** (**±**0.62)	**a**	**218.2** (**±**141.9)	**a**	—	—	**17.8** (**±**11.6)	**a**
	**THP1.0(T)**	**4.7** (**±**2.9)	**b**	—	—	**0.34** (**±**0.21)	**b**	**36.7** (**±**40.4)	**b**	—	—	**2.7** (**±**2.9)	**b**

^a^MLE to ACM/day was calculated from MLE to nicotine/day using the relationship between ACM and nicotine, determined from calibration smoking (see section 2.4.2).

^b^For IS1.0(T), MLE to nicotine per day was calculated by multiplying the average daily consumption e-liquid (mL) per day by the concentration of nicotine (mg/mL) in the cartridge.

^c^Different letters within a group indicate statistically significant differences (p < 0.05) between mean values.

There were no significant differences in MLE to ACM and nicotine between user groups 1 and 2 for IS1.0(T) and MLE to NFDPM and nicotine between user groups 1 and 3 for C651 (Table 5).

**Table 5:** Comparison of MLE to NFDPM/ACM and nicotine between user groups.

Product	User group	MLE to NFDPM (mg/session)		MLE to ACM (mg/session)		MLE to nicotine (mg/session)		MLE to NFDPM (mg/day)		MLE to ACM^a^ (mg/day)		MLE to nicotine^b^ (mg/day)	
		Mean (±SD)	Pr > (t)^c^	Mean (±SD)	Pr > (t)^c^	Mean (±SD)	Pr > (t)^c^	Mean (±SD)	Pr > (t)^c^	Mean (±SD)	Pr > (t)^c^	Mean (±SD)	Pr > (t)^c^
C651	**1**	**19.0** (**±**7.7)	**0.1201**	—	—	**1.55** (**±**0.63)	**0.1195**	**256.4** (**±**150.4)	**0.1471**	—	—	**20.9** (**±**12.3)	**0.1467**
	**3**	**16.7** (**±**7.6)				**1.36** (**±**0.62)		**218.2** (**±**141.9)				**17.8** (**±**11.6)	
IS1.0(T)	**1**	—	—	**180.1** (**±**137.8)	**0.2735**	**0.69** (**±**0.53)	**0.2698**	—	—	**1883.1** (**±**897.6)	**0.6693**	**7.3** (**±**3.5)	**0.6693**
	**2**			**209.2** (**±**157.4)		**0.80** (**±**0.60)				**1914.2** (**±**679.4)		**7.4** (**±**2.6)	

^a^MLE to ACM/day was calculated from MLE to nicotine/day using the relationship between ACM and nicotine, determined from calibration smoking (see section 2.4.2).

^b^For IS1.0(T), MLE to nicotine per day was calculated by multiplying the average daily consumption e-liquid (mL) per day by the concentration of nicotine (mg/mL) in the cartridge.

^c^Values > 0.05 indicate no statistically significant difference.

### Average daily consumption (ADC)

For both C651 and IS1.0(T), there were no significant differences in ADC when comparing the same product between different groups (Table 6).

**Table 6:** Comparison of participant’s ADC of study product consumables across user groups.

Product	User Group	Self-reported ADC of cigarettes at screening	ADC of study product consumables reported in consumption diary		Total ADC reported in consumption diary^c^	
			Mean (±SD)	Pr > (t)^a^	Mean (±SD)	Pr > (t)^a^
*C65*1	**1**	12.8 (**±**3.9)	**13.2** (**±**4.4)	0.4459	**13.5** (**±**4.3)	0.5989
	**3**	13.0 (**±**4.4)	**12.6** (**±**4.7)		**13.1** (**±**4.9)	
*IS1.0(T)*	**1**	12.8 (**±**3.9)	**1.5** (**±**0.7)^**b**^	0.6693	**5.0** (**±**3.5)	—
	**2**	—	**1.5** (**±**0.5)^**b**^		—	
*THP1.0(T)*	**3**	13.0 (**±**4.4)	**7.0** (**±**5.5)	—	**11.6** (**±**5.7)	—
*THS*2*.4(T)*	**1**	12.8 (**±**3.9)	**8.5** (**±**5.2)	—	**11.8** (**±**5.5)	—

^a^Values > 0.05 indicate no statistically significant difference.

^b^ADC of IS1.0(T) presented as mL of e-liquid consumed, calculated using number of cartridges consumed per day.

^c^Total ADC calculated using sum of number of study products (mL of e-liquid for IS1.0(T)) and number of non-study products consumed per day.

The ADC of cigarettes by groups 1 and 3 were higher than the respective ADCs of both THP consumables. When comparing across user groups, the consumption of THS2.4(T) consumables was higher than that of the THP1.0(T), whilst the total ADC values were similar.

The total ADCs for all groups were higher than the consumption of study products, suggesting that at least some users in all study groups were using non-study products during the home placements.

Within Groups 1 and 3, the ADC of C651 remained similar to that of cigarettes at screening. When using IS1.0(T), participants in Groups 1 and 2 reported significantly lower total ADCs throughout the study compared to at screening.

### Verification of optical NFDPM and nicotine estimates

**Figure 3:**
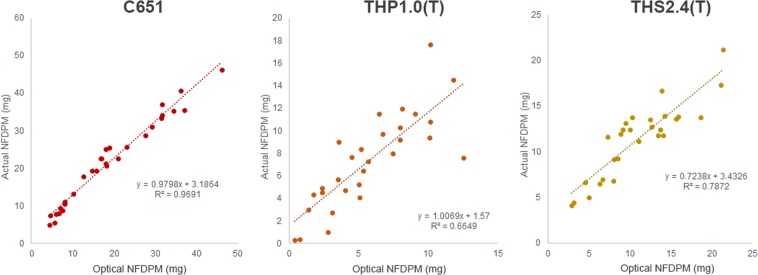
Comparison graphs of optical NFDPM vs. Actual NFDPM for C651, THP1.0(T) and THS2.4(T).

For the THPs and tobacco cigarette, correlations between the actual NFDPM and optical NFDPM were observed. *R*^2^ values were calculated as 0.9691 for C651, 0.6649 for THP1.0(T) and 0.7872 for THS2.4(T) (Fig. 3).

**Figure 4:**
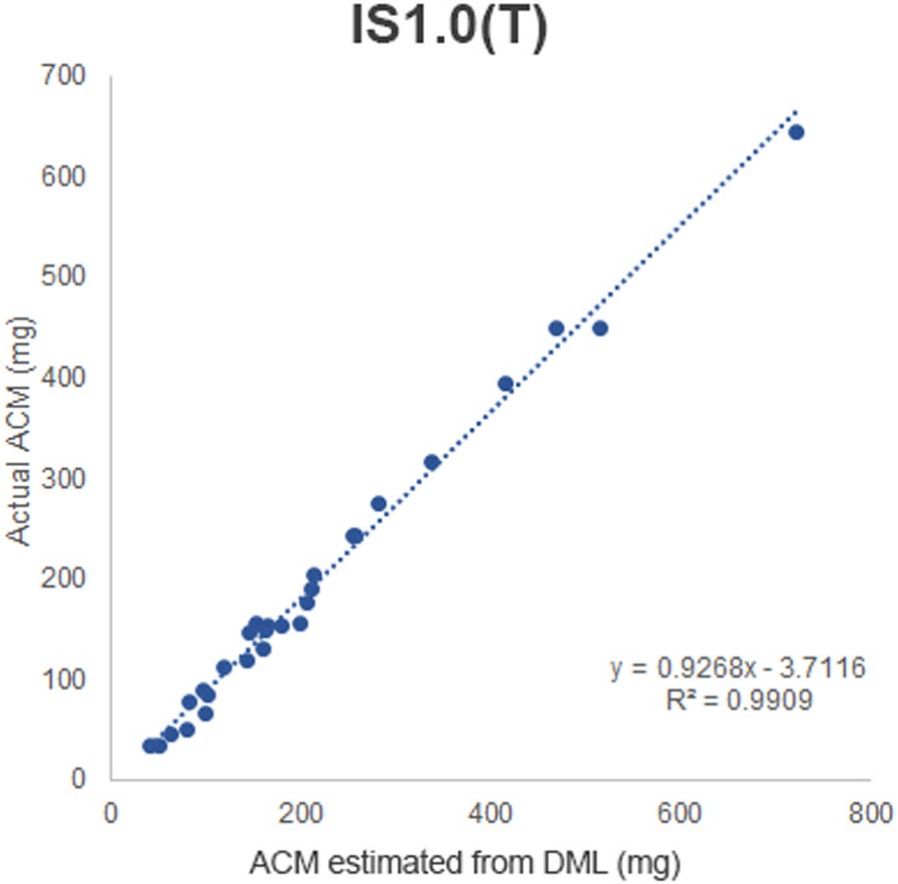
Comparison graph of ACM estimated from DML vs. Actual ACM for IS1.0(T).

Similarly, the e-cigarette (IS1.0(T)) displayed a strong correlation between DML and ACM, with an *R*^2^ value of 0.9909 (Fig. 4).

**Figure 5:**
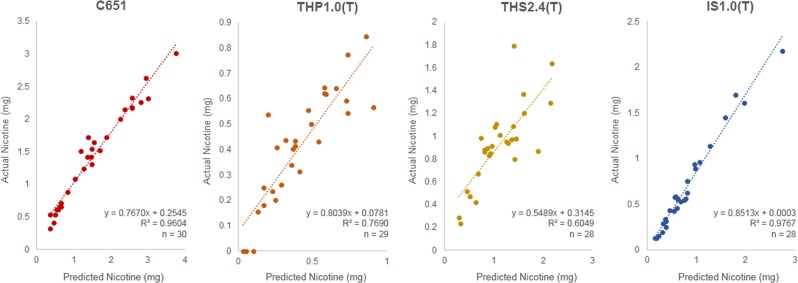
Comparison graphs of predicted Nicotine vs. Actual Nicotine for all products.

For the nicotine predictions, R^2^ values (Fig. 5) varied between 0.6053 and 0.9767 across the four products tested.

### Sensory questionnaire

Results from the sensory questionnaire are summarised in Table 7.

**Table 7:** Summary table for sensory scores from CLT and HUT surveys.

		*Central Location*						*Home Use*					
		Group 1		Group 2		Group 3		Group 1		Group 2		Group 3	
		C651	IS1.0(T)	THS2.4(T)	IS1.0(T)	C651	THP1.0(T)	C651	IS1.0(T)	THS2.4(T)	IS1.0(T)	C651	THP1.0(T)
Immediate smoke/aerosol delivery	*Magnitude Scale*^1^	A	B	B	—	A	B	A	B	B	—	A	B
	*Just Right’ Scale*^2^	JR	JR	LOW	LOW	JR	LOW	LOW	JR	LOW	LOW	JR	LOW
Draw effort	*Magnitude Scale*^1^	A	A	A	—	B	A	A	A	A	—	B	A
	*Just Right’ Scale*^2^	HIGH	JR	HIGH	HIGH	HIGH	HIGH	JR	JR	HIGH	HIGH	HIGH	HIGH
Mouthful	*Magnitude Scale*^1^	A	AB	B	—	A	B	A	A	B	—	A	B
	*Just Right’ Scale*^2^	JR	JR	LOW	JR	JR	LOW	LOW	JR	LOW	JR	JR	LOW
Irritation	*Magnitude Scale*^1^	B	A	B	—	A	A	B	A	B	—	B	A
	*Just Right’ Scale*^2^	JR	HIGH	JR	HIGH	JR	JR	JR	HIGH	JR	HIGH	JR	HIGH
Intensity of kick/hit	*Magnitude Scale*^1^	B	A	AB	—	A	A	B	A	AB	—	A	A
	*Just Right’ Scale*^2^	JR	HIGH	JR	HIGH	JR	JR	JR	HIGH	JR	HIGH	JR	HIGH
Taste - Likeability	*Magnitude Scale*^1^	A	B	B	—	A	B	A	B	B	—	A	B
	*Just Right’ Scale*^2^	—	—	—	—	—	—	—	—	—	—	—	—
Taste - Amount	*Magnitude Scale*^1^	A	AB	B	—	A	B	A	A	A	—	A	A
	*Just Right’ Scale*^2^	JR	JR	JR	JR	JR	JR	JR	JR	JR	HIGH	JR	JR
Overall likeability	*Magnitude Scale*^*1*^	A	B	B	—	A	B	A	B	B	—	A	B
	*Just Right’ Scale*^*2*^	—	—	—	—	—	—	—	—	—	—	—	—

^1^Different letters within a group indicate statistically significant differences (p < 0.05) between mean values.

^2^JR = No significant difference between mean score and score of 3 (Just Right), HIGH = Mean score associated with ‘Too High’ or ‘Significantly Too High’, LOW = Mean score associated with ‘Too Low’ or ‘Significantly Too Low’

For each sensory attribute, mean magnitude scores (1–7) are presented in Supplementary Tables S6-S13, alongside mean ‘just right’ scores (1–5).

## Discussion

Within the tobacco industry, recent advances have aimed to reduce the potential health risks associated with smoking. Products now available are designed to give consumers a similar sensation to smoking a traditional cigarette, whilst exposing users to significantly fewer tobacco-related toxicants or removing tobacco exposure altogether (e.g. e-cigarettes). The results from this study support the development of a reduced risk approach for THPs, suggesting that Italian consumer MLE to NFDPM and nicotine was significantly reduced for THP1.0(T) and THS2.4(T) compared to the tobacco cigarette (C651). MLE to nicotine was also significantly reduced for IS.0(T) compared to C651, however MLE to ACM cannot be directly compared with MLE to NFDPM from the other study products, due to fundamental differences in aerosol composition between cigarette smoke, THP aerosol and e-cigarette vapour^8,25^, with ACM measurements also including both water and nicotine. With regards to both THPs, NFDPM measurements are directly compared between cigarettes and THPs, however, the quantity and identity of the components contained within each are likely to differ significantly, as discussed later in the paper. It is also important to note that the water analysis for all products was based on widely used trapping and extraction procedures, such as those outlined in the International Standard ISO 4387. Ghosh and Jeannet^25^ previously suggested that for high water-content aerosols, such as those found with THP1.0(T) and THS2.4(T), this method may not account for all water released, leading to an overestimation of NFDPM, although further investigation is needed.

During the calibration stage of the study, it became apparent that the relationship between ACM and the SA7 optical response was relatively poor for IS1.0(T) compared to the other study products. It is possible that this may be due to fundamental differences in the nature of the aerosol (for example, optical response, particle size, mass and number) produced from these electronic nicotine delivery devices compared to tobacco cigarettes or THPs^26,27^. However, a strong relationship was observed between ACM and DML (R² = 0.9774), therefore, DML was used to estimate MLE. The results from this study demonstrate that DML is a better alternative to optical obscuration for measuring MLE to ACM and nicotine from IS1.0(T), further endorsed by the very strong correlations seen when duplicating the human records using a lab-based smoking machine.

Laboratory duplication of human records displayed strong relationships between predicted and actual nicotine and NFDPM or ACM across all products, suggesting that the methods used within this study are suitable for estimating consumer MLE to these compounds. Future studies may compare MLE to ACM nicotine across different vapour products, to confirm that these relationships hold true for a variety of different ENDS.

The puffing topography data collected in this study provides additional evidence suggesting that consumers are, on average, more likely to take larger puff volumes when using a THP compared to a cigarette or e-cigarette. With regards to IS1.0(T), mean puff volumes and puff durations were comparable to those seen for the cigarette (C651), however significantly higher puff numbers per session were recorded for IS1.0(T) (58.6 vs. 17.3). There were no statistically significant differences seen between Group 1 (smokers) and Group 2 (vapers or dual users), suggesting that a familiarisation period of 5-days was sufficient time to enable collection of consumer-representative data. Comparisons between this study and Gee *et al*.^18^ highlight the use-behaviour differences between Italian and Japanese consumers of tobacco and nicotine products. Italian smokers using THP1.0(T) were, on average, observed to take more puffs per session (15.4 ± 7.4 vs. 10.9 ± 5.6), lower mean puff volumes (46.6 mL ± 1 6.8 mL vs. 66.7 mL ± 23.7 mL) and longer intervals between puffs (11.1 s ± 5.8 s vs. 7.4 s ± 2.7 s). Aside from these differences, total puff volumes per stick were similar across smokers from the two populations (731.3 mL ± 437.6 mL vs 736.4 mL ± 415.8 mL), leading to similar gross exposure to NFDPM (4.7 mg ± 3.0 mg vs 5.2 mg ± 3.4 mL) and Nicotine (0.3 mg ± 0.2 mg vs 0.3 mg ± 0.2 mg) per stick.

Reported usage of non-study products by participants using C651 was negligible (on average less than 1 non-study consumable per home placement), whereas participants consumed a higher number of non-study products when using a THP (3–5 non-study consumables per home placement). It should be highlighted, however, that these numbers are significantly lower than the self-reported ADC of cigarettes at screening, suggesting an overall reduction in the number of cigarettes consumed whilst simultaneously using a THP. Moreover, the total ADCs when using these products remained significantly lower than for C651, further associating an overall decrease in daily consumption of tobacco products with the use of THP1.0(T) and THS2.4(T). Both MLE and consumption data relating to C651 from this study are consistent with a cigarette MLE filter-analysis study across a range of ISO tar yields in eight countries^28^.

At each visit to the central study location, participants were asked to complete a sensory questionnaire aimed at further explaining puffing topography data and building a more complete picture of the product use experience. Questions were based around use-behaviour and asked participants to score various attributes on both magnitude and ‘just-right’ scales for each product. When using the THPs, participants reported significantly lower mean *aerosol delivery and mouthful* scores compared to the tobacco cigarette. These attributes may help explain the higher mean puff volumes associated with THP use, as users would need to draw larger puffs in an attempt to obtain a satisfactory amount of aerosol from the consumables. *Taste likeability*, on average, scored significantly higher for C651 compared to the other products, although this may be at least partially linked to taste familiarisation as all participants using C651 were regular tobacco cigarette smokers.

Consumer use-behaviour and consumption data help give context to toxicological risk assessments and ensure that laboratory-based product testing is reflective of real-world consumers. A recent study by Forster *et al*.^8^ concluded that THP1.0(T) emissions are likely to contain, on average, >90.0% less TobReg 9 toxicants *per puff* when compared to a reference tobacco cigarette under laboratory conditions. When considering the significant differences in ADC and total puff volumes between THP1.0(T) and tobacco cigarettes (also observed by Gee *et al*.^18^), it may be suggested that the reduction in consumer exposure to these toxicants is further amplified when comparing exposure *per day* rather than *per stick*. Furthermore, the differences seen between IS1.0(T) and C651 puffing topography and consumption data further support the need for assessing toxicological risk through overall exposure *per day* when conducting cross-category evaluations. Although exposure to NFDPM is reduced, it should be noted that no assumptions relating to overall health risk can be drawn from this alone.

Generally, the machine puffing regimes used for pre-clinical laboratory measurements were broadly supported by the puffing topography measurements, despite the differences between products. This was further supported for THP1.0(T) where the 95th percentile MLE for optical NFDPM in the actual use study was in line with the mHCI (55 mL puff over 2 s every 30 seconds^29^, with no vent blocking^18^) machine measured NFDPM yield (Table S4). The average MLE to ACM for IS1.0(T) was also in line with the existing 55/3/30 machine testing regime^30^ (Table S5).

## Conclusions

The e-cigarette (IS1.0(T)) and both THPs (THP1.0(T), THS2.4(T)) were consumed in a manner that supports the reduced exposure hypothesis, which in turn has the potential to support any further toxicological risk assessment of these tobacco and nicotine products. Overall product consumption was reduced compared to tobacco cigarettes and, where comparable, MLE to both nicotine and NFDPM was significantly lower. Therefore, a combination of reduced toxicant emissions, topography and consumption data supports the overall reduction in consumer toxicant exposure when using these next-generation products over cigarettes. Consumer usage sessions were, on average, considerably longer with higher puff numbers for IS1.0(T) compared to the other study products, however this was likely due to the stick-based products having more restricted use-duration. Differences seen between sensory scores for each of the product categories may be attributed to fundamental differences in design and mode of operation resulting in different characteristics of the aerosol generated.

## Supplementary information

Supplementary Dataset 1

## Data Availability

The authors confirm that the data within the article and its supplementary materials are supportive of the study findings.
