# Supplementary material for: A cross-category puffing topography, mouth level exposure and consumption study among Italian users of tobacco and nicotine products
Source: Sci Rep. 2020 Jan 8;10:12. doi: 10.1038/s41598-019-55410-5 (PMC6949288; doi:10.1038/s41598-019-55410-5)
Supplement: Supplementary file 1 — Supplementary Dataset 1 [file 41598_2019_55410_MOESM1_ESM.docx]

**Supplementary Tables**

*A cross-category puffing topography, mouth level exposure and consumption study among Italian users of tobacco and nicotine products*

Joshua Jones, Sandra Slayford, Adam Gray, Kathryn Brick, Krishna Prasad*, Christopher Proctor

**Supplementary Tables**

**Table S1: Standard optical NFDPM/Nicotine calibration smoking regimes for tobacco products**

| **Standard Calibration Puffing Regimes (C651, THP1.0(T), THS2.4(T))** | | |
| --- | --- | --- |
| **Volume (mL)** | **Puff Duration (s)** | **Puff Frequency (s)** |
| 20 | 1.0 | 30 |
| 20 | 1.5 | 30 |
| 20 | 2.0 | 30 |
| 40 | 1.0 | 30 |
| 40 | 1.5 | 30 |
| 40 | 2.0 | 30 |
| 60 | 1.0 | 30 |
| 60 | 1.5 | 30 |
| 60 | 2.0 | 30 |
| 80 | 1.0 | 30 |
| 80 | 1.5 | 30 |
| 80 | 2.0 | 30 |
| 120 | 3.5 | 30 |

**Table S2: Standard optical NFDPM/Nicotine calibration smoking regimes for IS1.0(T)**

| **Standard Calibration Puffing Regimes (IS1.0(T))** | | |
| --- | --- | --- |
| **Volume (mL)** | **Puff Duration (s)** | **Puff Frequency (s)** |
| 40 | 1.5 | 30 |
| 40 | 2.0 | 30 |
| 80 | 2.0 | 30 |
| 80 | 3.0 | 30 |
| 80 | 4.0 | 30 |
| 120 | 2.0 | 30 |
| 120 | 3.0 | 30 |
| 120 | 4.0 | 30 |
| 120 | 6.0 | 30 |

**Table S3: Additional optical NFDPM/Nicotine calibration smoking regimes for C651**

| **Additional Calibration Puffing Regimes (C651)** | | |
| --- | --- | --- |
| **Volume (mL)** | **Puff Duration (s)** | **Puff Frequency (s)** |
| 20 | 1.0 | 10 |
| 40 | 1.0 | 10 |
| 60 | 1.5 | 10 |
| 80 | 1.5 | 10 |

**Table S4: Comparison of 95^th^ percentile MLE data with results from machine smoking (THP1.0(T), THS2.4(T) and C651)**

| **Product** | **95^th^ Percentile MLE to NFDPM (mg)** | **Mean (± SD) NFDPM from mHCI machine smoking (mg)** | **Mean (± SD) NFDPM from HCI machine smoking (mg)** |
| --- | --- | --- | --- |
| THP1.0(T) | 10.0 | 11.1 ± 2.1 | - |
| THS2.4(T) | 16.3 | 15.7 ± 2.3 | - |
| C651 (Group 1) | 32.2 | - | 29.8 ± 2.4 |
| C651 (Group 3) | 26.5 |  |  |

**Table S5: Comparison of mean MLE data with results from machine smoking (IS1.0(T))**

| **Product** | **Mean (± SD) MLE to ACM (mg)** | **Mean (± SD) ACM from 55/3/30 machine smoking (mg)** |
| --- | --- | --- |
| IS1.0(T) (Group 1) | 180.1 ± 137.8 | 227.0 ± 9.9 |
| IS1.0(T) (Group 2) | 209.2 ± 157.4 |  |

**Table S6: Immediate smoke/aerosol delivery scores for CLT and HUT surveys**

| **Immediate smoke/aerosol delivery**  *Question: How would you rate the Immediate smoke delivery, that is, the extent to which the first few puffs of the cigarette provides a desired amount of smoke in your mouth?* | | | | | | | | | | | | |
| --- | --- | --- | --- | --- | --- | --- | --- | --- | --- | --- | --- | --- |
| **Product** | ***Mean Scores (1-7)*** (± SD)  [Tukey’s Ranking^1^]  ***1=Lowest 🡪 7=Highest*** | | | | | | ***Mean JR Scale Scores (1-5)*** (± SD)  ***1=Too low; 2=Slightly too low; 3=Just right; 4=Slightly too high; 5=Too high*** | | | | | |
|  | ***Central Location*** | | | ***Home Use*** | | | ***Central Location*** | | | ***Home Use*** | | |
|  | Group 1 | Group 2 | Group 3 | Group 1 | Group 2 | Group 3 | Group 1 | Group 2 | Group 3 | Group 1 | Group 2 | Group 3 |
| C651 | **5.4 (**± 1.3) [a] | **-** | **5.0 (**± 1.3) [a] | **5.5 (**± 1.5) [a] | **-** | **5.4 (**± 1.1) [a] | **3.0 (**± 0.5) | **-** | **3.0 (**± 0.6) | **2.8* (**± 0.4) | **-** | **3.0 (**± 0.3) |
| IS1.0(T) | **4.3 (**± 1.6) [b] | **4.3 (**± 1.7) | **-** | **4.3 (**± 1.8) [b] | **4.4 (**± 1.9) | **-** | **2.8 (**± 0.7) | **2.7* (**± 1.0) | **-** | **3.0 (**± 1.0) | **2.7* (**± 1.1) | **-** |
| THP1.0(T) | **-** | **-** | **3.3 (**± 1.6) [b] | **-** | **-** | **3.0 (**± 1.6) [b] | **-** | **-** | **2.2* (**± 0.7) | **-** | **-** | **2.2* (**± 1.0) |
| THS2.4(T) | **3.7 (**± 1.7) [b] | **-** | **-** | **3.7 (**± 1.7) [b] | **-** | **-** | **2.5* (**± 0.8) | **-** | **-** | **2.4* (**± 0.7) | **-** | **-** |
| **^*^Statistically significant difference (p > 0.05) between the product score and score associated with ‘Just Right’ (3) ^1^Different letters within a group indicate statistically significant differences (p<0.05) between mean values** | | | | | | | | | | | | |

**Table S7: Mean Draw Effort Scores for CLT and HUT surveys**

| **Draw Effort**  *Question: How would you rate the draw effort, which is the amount of effort needed whilst drawing on the cigarette to get a satisfactory puff of smoke?* | | | | | | | | | | | | | |
| --- | --- | --- | --- | --- | --- | --- | --- | --- | --- | --- | --- | --- | --- |
| **Product** | ***Mean Scores (1-7)*** (± SD) [Tukey’s Ranking^1^]  ***1=Lowest 🡪 7=Highest*** | | | | | | ***Mean JR Scale Scores (1-5)*** (± SD)  ***1=Too low; 2=Slightly too low; 3=Just right; 4=Slightly too high; 5=Too high*** | | | | | | |
|  | ***Central Location*** | | | ***Home Use*** | | | ***Central Location*** | | | ***Home Use*** | | | |
|  | Group 1 | Group 2 | Group 3 | Group 1 | Group 2 | Group 3 | Group 1 | Group 2 | Group 3 | Group 1 | Group 2 | Group 3 |  |
| C651 | **3.5 (**± 1.7) [a] | **-** | **3.8 (**± 1.5) [b] | **3.6 (**± 1.6) [a] | **-** | **3.7 (**± 1.4) [b] | **3.1* (**± 0.3) | **-** | **3.2* (**± 0.6) | **3.0 (**± 0.4) | **-** | **3.1* (**± 0.3) |  |
| IS1.0(T) | **4.2 (**± 1.6) [a] | **4.2 (**± 1.9) | **-** | **3.6 (**± 1.9) [a] | **3.7 (**± 1.9) | **-** | **3.2 (**± 0.8) | **3.4* (**± 1.0) | **-** | **3.0 (**± 0.9) | **3.4* (**± 0.9) | **-** |  |
| THP1.0(T) | **-** | **-** | **4.9 (**± 1.6) [a] | **-** | **-** | **4.9 (**± 1.7) [a] | **-** | **-** | **3.6* (**± 0.8) | **-** | **-** | **3.7* (**± 1.0) |  |
| THS2.4(T) | **4.1 (**± 1.7) [a] | **-** | **-** | **4.3 (**± 1.9) [a] | **-** | **-** | **3.3* (**± 0.9) | **-** | **-** | **3.5* (**± 0.9) | **-** | **-** |  |
| **^*^Statistically significant difference (p > 0.05) between the product score and score associated with ‘Just Right’ (3)**  **^1^Different letters within a group indicate statistically significant differences (p<0.05) between mean values** | | | | | | | | | | | | |  |

**Table S8: Mean Mouthful scores for CLT and HUT surveys**

| **Mouthful**  *Question: How would you rate the amount of smoke filling the mouth on normal puff taking?* | | | | | | | | | | | | | |
| --- | --- | --- | --- | --- | --- | --- | --- | --- | --- | --- | --- | --- | --- |
| **Product** | ***Mean Scores (1-7)*** (± SD) [Tukey’s Ranking^1^]  ***1=Lowest 🡪 7=Highest*** | | | | | | ***Mean JR Scale Scores (1-5)*** (± SD)  ***1=Too low; 2=Slightly too low; 3=Just right; 4=Slightly too high; 5=Too high*** | | | | | | |
|  | ***Central Location*** | | | ***Home Use*** | | | ***Central Location*** | | | ***Home Use*** | | | |
|  | Group 1 | Group 2 | Group 3 | Group 1 | Group 2 | Group 3 | Group 1 | Group 2 | Group 3 | Group 1 | Group 2 | Group 3 |  |
| C651 | **4.8 (**± 1.0) [a] | **-** | **4.5 (**± 1.2) [a] | **4.4 (**± 1.0) [a] | **-** | **4.6 (**± 1.0) [a] | **3.0 (**± 0.4) | **-** | **3.0 (**± 0.6) | **2.9* (**± 0.3) | **-** | **2.9 (**± 0.4) |  |
| IS1.0(T) | **4.3 (**± 1.3) [ab] | **4.0 (**± 1.5) | **-** | **4.3 (**± 1.6) [a] | **4.2 (**± 1.6) | **-** | **3.0 (**± 0.8) | **2.7 (**± 0.9) | **-** | **3.0 (**± 1.0) | **2.8 (**± 0.9) | **-** |  |
| THP1.0(T) | **-** | **-** | **3.2 (**± 1.3) [b] | **-** | **-** | **3.2 (**± 1.6) [b] | **-** | **-** | **2.3* (**± 0.7) | **-** | **-** | **2.3* (**± 0.8) |  |
| THS2.4(T) | **3.8 (**± 1.3) [b] | **-** | **-** | **3.5 (**± 1.5) [b] | **-** | **-** | **2.7* (**± 0.8) | **-** | **-** | **2.4* (**± 0.8) | **-** | **-** |  |
| \| **^*^Statistically significant difference (p > 0.05) between the product score and score associated with ‘Just Right’ (3)**  **^1^Different letters within a group indicate statistically significant differences (p<0.05) between mean values** \| \| --- \| | | | | | | | | | | | | |  |

**Table S9: Mean Irritation scores for CLT and HUT surveys**

| **Irritation**  *Question: How would you rate the level of irritation, that is, the tingling and prickling physical sensation you felt in your mouth, nose or throat while inhaling or exhaling?* | | | | | | | | | | | | | |
| --- | --- | --- | --- | --- | --- | --- | --- | --- | --- | --- | --- | --- | --- |
| **Product** | ***Mean Scores (1-7)*** (± SD) [Tukey’s Ranking^1^]  ***1=Lowest 🡪 7=Highest*** | | | | | | ***Mean JR Scale Scores (1-5)*** (± SD)  ***1=Too low; 2=Slightly too low; 3=Just right; 4=Slightly too high; 5=Too high*** | | | | | | |
|  | ***Central Location*** | | | ***Home Use*** | | | ***Central Location*** | | | ***Home Use*** | | | |
|  | Group 1 | Group 2 | Group 3 | Group 1 | Group 2 | Group 3 | Group 1 | Group 2 | Group 3 | Group 1 | Group 2 | Group 3 |  |
| C651 | **2.9 (**± 1.8) [b] | **-** | **3.3 (**± 1.4) [a] | **2.3 (**± 1.7) [b] | **-** | **2.6 (**± 1.7) [b] | **3.0 (**± 0.6) | **-** | **3.0 (**± 0.7) | **2.9 (**± 0.6) | **-** | **2.9 (**± 0.8) |  |
| IS1.0(T) | **4.1 (**± 2.0) [a] | **4.5 (**± 1.8) | **-** | **3.8 (**± 2.3) [a] | **4.1 (**± 2.0) | **-** | **3.5* (**± 0.9) | **3.6* (**± 0.9) | **-** | **3.5* (**± 1.1) | **3.4* (**± 1.0) | **-** |  |
| THP1.0(T) | **-** | **-** | **3.6 (**± 1.9) [a] | **-** | **-** | **3.5 (**± 2.2) [a] | **-** | **-** | **3.1 (**± 1.1) | **-** | **-** | **3.4* (**± 1.1) |  |
| THS2.4(T) | **3.4 (**± 2.0) [ab] | **-** | **-** | **3.0 (**± 1.8) [ab] | **-** | **-** | **3.1 (**± 1.1) | **-** | **-** | **2.8 (**± 1.1) | **-** | **-** |  |
| \| **^*^Statistically significant difference (p > 0.05) between the product score and score associated with ‘Just Right’ (3)**  **^1^Different letters within a group indicate statistically significant differences (p<0.05) between mean values** \| \| --- \| | | | | | | | | | | | | |  |

**Table S10: Mean Intensity of kick/hit scores for CLT and HUT surveys**

| **Intensity of kick/hit**  *Question: How would you rate the intensity of kick/hit on the throat – the short, sharp, momentary “kick” or “hit” sensation you may have felt as the smoke goes down your throat?* | | | | | | | | | | | | | |
| --- | --- | --- | --- | --- | --- | --- | --- | --- | --- | --- | --- | --- | --- |
| **Product** | ***Mean Scores (1-7)*** (± SD) [Tukey’s Ranking^1^]  ***1=Lowest 🡪 7=Highest*** | | | | | | ***Mean JR Scale Scores (1-5)*** (± SD)  ***1=Too low; 2=Slightly too low; 3=Just right; 4=Slightly too high; 5=Too high*** | | | | | | |
|  | ***Central Location*** | | | ***Home Use*** | | | ***Central Location*** | | | ***Home Use*** | | | |
|  | Group 1 | Group 2 | Group 3 | Group 1 | Group 2 | Group 3 | Group 1 | Group 2 | Group 3 | Group 1 | Group 2 | Group 3 |  |
| C651 | **3.4 (**± 1.8) [b] | **-** | **3.8 (**± 1.3) [a] | **3.1 (**± 1.5) [b] | **-** | **3.8 (**± 1.3) [a] | **3.0 (**± 0.4) | **-** | **3.1 (**± 0.6) | **3.0 (**± 0.5) | **-** | **3.0 (**± 0.5) |  |
| IS1.0(T) | **4.2 (**± 1.8) [a] | **4.9 (**± 1.3) | **-** | **4.0 (**± 1.9) [a] | **4.7 (**± 1.6) | **-** | **3.3* (**± 0.9) | **3.5* (**± 0.8) | **-** | **3.3* (**± 1.0) | **3.4* (**± 0.9) | **-** |  |
| THP1.0(T) | **-** | **-** | **3.9 (**± 1.8) [a] | **-** | **-** | **4.1 (**± 1.8) [a] | **-** | **-** | **3.2 (**± 1.0) | **-** | **-** | **3.3* (**± 1.0) |  |
| THS2.4(T) | **3.6 (**± 1.7) [ab] | **-** | **-** | **3.3 (**± 1.6) [ab] | **-** | **-** | **3.0 (**± 0.9) | **-** | **-** | **3.0 (**± 0.8) | **-** | **-** |  |
| \| **^*^Statistically significant difference (p > 0.05) between the product score and score associated with ‘Just Right’ (3)**  **^1^Different letters within a group indicate statistically significant differences (p<0.05) between mean values** \| \| --- \| | | | | | | | | | | | | |  |

**Table S11: Mean Taste Likeability Scores for CLT and HUT surveys**

| **Taste – Likeability**  *Question: Now considering only the taste of the cigarettes, can you tell me how much do you like the taste of these cigarettes?* | | | | | | |
| --- | --- | --- | --- | --- | --- | --- |
| **Product** | ***Mean Scores (1-7)*** (± SD) [Tukey’s Ranking^1^]  ***1=Lowest 🡪 7=Highest*** | | | | | |
|  | ***Central Location*** | | | ***Home Use*** | | |
|  | Group 1 | Group 2 | Group 3 | Group 1 | Group 2 | Group 3 |
| C651 | **5.2 (**± 1.3) [a] | **-** | **5.1 (**± 1.6) [a] | **5.3 (**± 1.5) [a] | **-** | **5.3 (**± 1.5) [a] |
| IS1.0(T) | **3.8 (**± 2.0) [b] | **4.2 (**± 1.7) | **-** | **3.6 (**± 2.1) [b] | **4.3 (**± 2.0) | **-** |
| THP1.0(T) | **-** | **-** | **2.8 (**± 2.0) [b] | **-** | **-** | **2.5 (**± 1.8) [b] |
| THS2.4(T) | **3.4 (**± 2.0) [b] | **-** | **-** | **3.4 (**± 1.9) [b] | **-** | **-** |
| **^1^Different letters within a group indicate statistically significant differences (p<0.05) between mean values** | | | | | | |

**Table S12: Mean Taste Amount Scores for CLT and HUT surveys**

| **Taste – Amount**  *Question: How would you rate the amount of taste or flavour of the cigarette, irrespective of whether you like the flavour or not?* | | | | | | | | | | | | | |
| --- | --- | --- | --- | --- | --- | --- | --- | --- | --- | --- | --- | --- | --- |
| **Product** | ***Mean Scores (1-7)*** (± SD) [Tukey’s Ranking^1^]  ***1=Lowest 🡪 7=Highest*** | | | | | | ***Mean JR Scale Scores (1-5)*** (± SD)  ***1=Too low; 2=Slightly too low; 3=Just right; 4=Slightly too high; 5=Too high*** | | | | | | |
|  | ***Central Location*** | | | ***Home Use*** | | | ***Central Location*** | | | ***Home Use*** | | | |
|  | Group 1 | Group 2 | Group 3 | Group 1 | Group 2 | Group 3 | Group 1 | Group 2 | Group 3 | Group 1 | Group 2 | Group 3 |  |
| C651 | **5.0 (**± 1.2) [a] | **-** | **4.6 (**± 1.3) [a] | **4.7 (**± 1.4) [a] | **-** | **4.8 (**± 1.2) [a] | **3.0 (**± 0.5) | **-** | **3.0 (**± 0.6) | **2.9 (**± 0.5) | **-** | **3.0 (**± 0.5) |  |
| IS1.0(T) | **4.4 (**± 1.7) [ab] | **4.7 (**± 1.3) | **-** | **4.3 (**± 1.7) [a] | **4.9 (**± 1.3) | **-** | **3.1 (**± 1.0) | **3.2 (**± 0.7) | **-** | **3.1 (**± 1.1) | **3.3* (**± 0.8) | **-** |  |
| THP1.0(T) | **-** | **-** | **4.0 (**± 1.8) [b] | **-** | **-** | **4.4 (**± 1.9) [a] | **-** | **-** | **3.0 (**± 1.1) | **-** | **-** | **3.1 (**± 1.1) |  |
| THS2.4(T) | **4.2 (**± 1.4) [b] | **-** | **-** | **4.3 (**± 1.8) [a] | **-** | **-** | **3.0 (**± 0.8) | **-** | **-** | **3.0 (**± 1.0) | **-** | **-** |  |
| \| **^*^Statistically significant difference (p > 0.05) between the product score and score associated with ‘Just Right’ (3)**  **^1^Different letters within a group indicate statistically significant differences (p<0.05) between mean values** \| \| --- \| | | | | | | | | | | | | |  |

**Table S13: Mean Likeability Scores for CLT and HUT surveys**

| **Overall Likeability**  *Question: Can you tell me how much do you like this product?* | | | | | | |
| --- | --- | --- | --- | --- | --- | --- |
| **Product** | ***Mean Scores (1-7)*** (± SD) [Tukey’s Ranking^1^]  ***1=Lowest 🡪 7=Highest*** | | | | | |
|  | ***Central Location*** | | | ***Home Use*** | | |
|  | Group 1 | Group 2 | Group 1 | Group 1 | Group 2 | Group 3 |
| C651 | **5.3 (**± 1.2) [a] | **-** | **5.2 (**± 1.4) [a] | **5.6 (**± 1.2) [a] | **-** | **5.6 (**± 1.2) [a] |
| IS1.0(T) | **3.6 (**± 1.9) [b] | **4.4 (**± 1.4) | **-** | **3.5 (**± 1.8) [b] | **4.3 (**± 1.7) | **-** |
| THP1.0(T) | **-** | **-** | **3.1 (**± 1.9) [b] | **-** | **-** | **2.8 (**± 1.6) [b] |
| THS2.4(T) | **3.6 (**± 1.9) [b] | **-** | **-** | **3.7 (**± 1.7) [b] | **-** | **-** |
| **^1^Different letters within a group indicate statistically significant differences (p<0.05) between mean values** | | | | | | |
